# Sex differences in a mouse model of radiation-induced cardiotoxicity

**DOI:** 10.21203/rs.3.rs-10020888/v1

**Published:** 2026-06-24

**Authors:** Emily R. Whelan, Evan E. Perona, Jessica J. Fliess, S. Christian Kocsis, Gabriel J. Weigel, Elizabeth J. McCabe, Charwan Hamilton, Damian N. Di Florio, Varsini Balamurugan, Danielle J. Beetler, Logan P. Macomb, Max W. Strandes, Frances C. Wilson, Emma F. Murphy, Ashley A. Darakjian, Molly M. Watkins, Alayna M. Puls, Cameron J. Hartmoyer, Natalie Bonvie-Hill, Sami Khatib, Chris J. McLeod, Jordan C. Ray, Michael D. Story, Bradford S. Hoppe, DeLisa Fairweather

**Affiliations:** Mayo Clinic in Florida; Mayo Clinic in Florida; Mayo Clinic in Florida; Mayo Clinic in Florida; Mayo Clinic in Florida; Mayo Clinic in Florida; Mayo Clinic in Florida; Mayo Clinic in Florida; Mayo Clinic in Florida; Mayo Clinic in Florida; Mayo Clinic in Florida; Mayo Clinic in Florida; Mayo Clinic in Florida; Mayo Clinic in Florida; Mayo Clinic in Florida; Mayo Clinic in Florida; Mayo Clinic in Florida; Mayo Clinic in Florida; Mayo Clinic in Florida; Mayo Clinic in Florida; Mayo Clinic in Florida; Mayo Clinic in Florida; Mayo Clinic in Florida; Mayo Clinic in Florida; Mayo Clinic in Florida

**Keywords:** radiation-induced heart disease, sex differences, vascular damage, mitochondrial dysfunction, inflammation, fibrosis, dilated cardiomyopathy

## Abstract

**Background:**

Radiation-induced heart disease (RIHD) may develop months to years following radiation therapy for cancers near the heart such as lung or breast cancer. Several mouse models of RIHD exist to study the pathogenesis of disease, but to our knowledge no studies have examined whether sex differences exist in RIHD in mice.

**Methods:**

Female and male BALB/cJ mice received one whole heart dose of 22 Gray (Gy) radiation via a C5 collimator vs. sham controls that received 0 Gy, anesthesia and were placed in the X-ray machine for the same length of time. Mechanisms of cardiotoxicity were examined at 24 h (innate cardiotoxicity), day 10 (acute cardiotoxicity) and day 35 (chronic cardiotoxicity) post irradiation.

**Results:**

Females and males developed dsDNA breaks from radiation at day 10 but females had more damage than males (*p* < 0.001). Vessel area and cardiac hypertrophy were increased by radiation at day 10 and 35 but were higher in males than females at day 35 (*p* < 0.001, *p* = 0.001, respectively). Bulk RNA sequencing of the heart at day 10 revealed that females and males developed similar gene pathway responses to radiation by upregulating cellular repair pathways and downregulated mitochondrial function. However, females upregulated the master regulator of mitochondrial function, Pgc1α (*p* = 0.0001), suggesting improved mitochondrial repair in females. By day 35, males developed heart failure with reduced ejection fraction (HFrEF), inflammatory dilated cardiomyopathy (iDCM) and fibrosis while females developed heart failure with preserved ejection fraction (HFpEF) without inflammation or fibrosis.

**Conclusions:**

We observed sex differences in nearly every cardiac parameter that we examined following radiation exposure. BALB/c males exposed to 22 Gy radiation developed HFrEF and iDCM by day 35 while females developed HFpEF. Further research is needed to better understand mechanisms driving sex differences in RIHD using translational animal models.

## Plain English summary

Radiation therapy used to treat cancers near the heart, such as breast and lung cancer, can sometimes cause heart damage months or years later, a condition known as radiation-induced heart disease (RIHD). Although mouse models are commonly used to study RIHD, little is known about whether males and females respond differently to radiation exposure. In this study, male and female BALB/cJ mice received a single radiation dose directed at the heart and were compared with non-irradiated control mice. Researchers examined early and later effects of radiation over a 35-day period. Both sexes experienced radiation-induced DNA damage in heart cells, but females showed greater DNA damage than males. Radiation also increased blood vessel size and heart enlargement in both sexes, although these changes were more pronounced in males. Gene expression analysis showed that males and females activated many of the same repair pathways after radiation and both showed reduced mitochondrial function. However, females increased expression of Pgc1α, a key regulator of mitochondrial health, suggesting they may have a greater ability to repair mitochondrial damage. Despite these similarities, the long-term cardiac outcomes differed substantially between sexes. By 35 days after radiation exposure, males developed inflammatory dilated cardiomyopathy, heart fibrosis, and heart failure with reduced ejection fraction (HFrEF). In contrast, females developed heart failure with preserved ejection fraction (HFpEF) without evidence of inflammation or fibrosis. These findings demonstrate important sex-specific differences in the cardiac response to radiation and highlight the need for further research to understand the biological mechanisms underlying these differences.

## Introduction

Radiation therapy is a common treatment for patients with thoracic malignancies such as breast cancer, lung cancer, Hodgkin’s lymphoma, and mediastinal lymphoma. Although low doses are used to prevent collateral damage to the heart when irradiating thoracic cancers, patients receiving radiation therapy are at risk of developing radiation-induced heart disease (RIHD) months to years later [1, 2]. RIHD can present clinically as cardiomyopathy/dilated cardiomyopathy (DCM), acute coronary syndrome with vascular damage, congestive heart failure, pericarditis, myocardial fibrosis, conduction disorders, and/or valvular disease [3–5]. RIHD is most often a diagnosis of exclusion, and for this reason is likely to be underdiagnosed [6]. Thus, there is a need to identify early diagnostic biomarkers that predict the risk of cardiomyopathy following radiation therapy for earlier diagnosis.

Men have a higher overall prevalence of cardiovascular diseases (CVDs) than women in the US (52.4% vs. 44.8%) [7]. Sex differences exist in individual CVS with most occurring in men like hypertension, atherosclerosis, myocarditis and DCM, while only a few CVDs have been found to occur more often in women like and cardiovascular-kidney-metabolic syndrome [8–10]. Sex differences also exist in heart failure (HF), with males primarily developing HF with reduced ejection fraction (HFrEF) while females are more likely to develop HF with preserved EF (HFpEF) [9, 11, 12]. Khalid et al. conducted a meta-analysis of 10 studies of 13,975 patients following radiation therapy, 41% females and 59% males, and found a higher RIHD prevalence and mortality in females compared to males [13]. Interestingly, they found that mortality from RIHD was higher in elderly women in their analysis [13]. Sex hormones and chromosomes promote sex differences in cardiac physiology and the immune response that drive cardiopathology [8, 9, 14, 15]. Further research is needed to better understand whether sex differences exist in RIHD.

Numerous animal models have examined radiation-induced cardiotoxicity using C57BL/6 males [16–19] or females [20–23]. However, few models have compared sex differences in RIHD. Andruska et al. examined whether sex differences exist in male vs. female inbred Dahl SS rats using 24 Gray (Gy) in a single fraction to the whole heart using a 1.5 cm collimator [24]. They found that 100% of females developed pericardial effusion and heart failure while only 14% of males developed this form of cardiomyopathy [24]. They identified that collateral damage to the lungs was greater in females because of their smaller heart size and when they increased the size of the collimator to 2.0 cm in males there was no sex difference in pericardial effusion or mortality by sex. To our knowledge, there are no studies examining sex differences in RIDH in mice. In this study we used moderately low dose radiation (i.e., 22 Gy) to the whole heart with very low lung collateral damage to study sex differences in RIHD.

## Results

### Cardiotoxicity model

**Figure 1:**
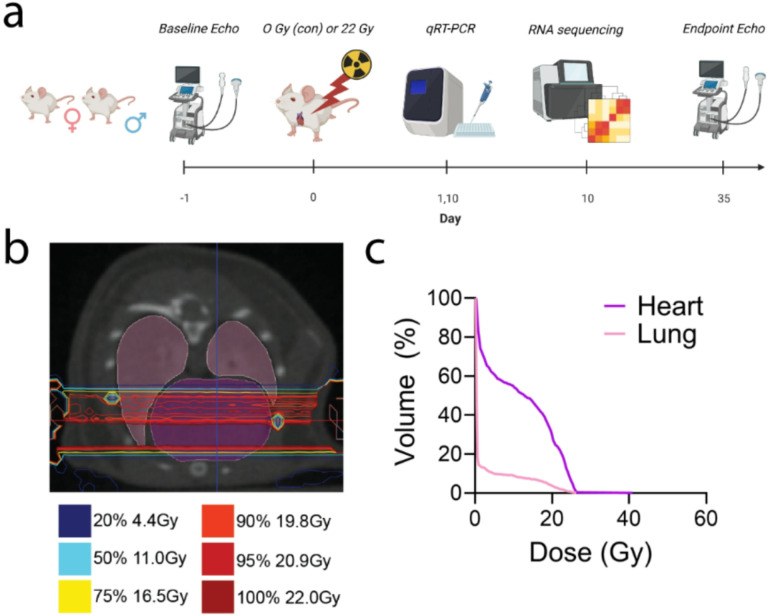
Cardiotoxicity model. **a)** Experimental timeline for male and female BALB/c mice. **b)** A representative plan for whole heart radiation using 22 Gy for male and female BALB/c mice showing the axial section. The heart (purple) was irradiated using a parallel opposed method in the axillary region. Mice were placed in a feet-first prone position in relation to the gantry which was equipped with a C5 collimator. The isocenter of radiation is aimed medial to the left and right ventricles and superior to the apex of the heart. **c)** Representative image of the dose-volume histogram (DVH) showing the percentage of heart (purple) and lung (pink) receiving 22 Gy radiation.

BALB/c mice were chosen for this study because C57BL mice are resistant to developing cardiomyopathy and do not display sex differences [8, 9, 25–28]. Female and male BALB/c mice were randomly assigned to receive 0 Gy (control) or 22 Gy based on preliminary data indicating that this was an effective dose (data not shown). Based on previous experience with murine models of cardiomyopathy,[8, 9, 29, 30] three time points were selected as endpoints: 24 hours (h) (innate cardiotoxicity), day 10 (acute cardiotoxicity) and day 35 (chronic cardiotoxicity/cardiomyopathy) (Fig. 1a). At 24 h we confirmed the presence of radiation damage by measuring γH2A.x and performed. Mice received a baseline echo on day − 1 and an endpoint echo at day 35 to detect cardiomyopathy (Fig. 1a). qRT-PCR was performed at 24 h and day 10 and 35 after radiation.

Figure 1b shows a representative treatment plan image of whole heart radiation aimed medial to the left and right ventricles and superior to the apex of the heart. A representative image of the dose-volume histogram (DVH) showing the percentage of heart (purple) and lung (pink) receiving 22 Gy radiation is shown in Fig. 1c, demonstrating very low collateral damage to the lungs.

#### Acute cardiotoxicity greater in females

**Figure 2:**
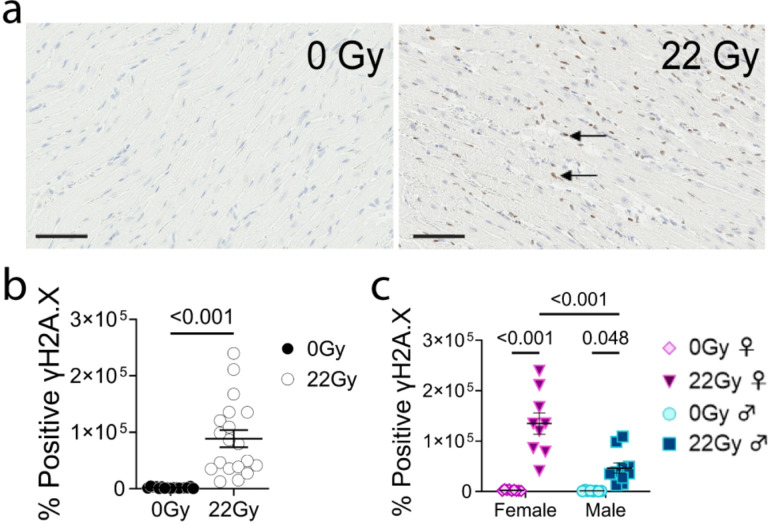
Sex differences in dsDNA breaks at day 10 post radiation. **a)** Representative images of gH2A.x IHC staining of 0 and 22 Gy (bars = 200μm). Positive staining is brown on a blue background (arrows). dsDNA breaks were quantified at day 10 from gH2A.x IHC and shown as the percent positive normalized to the total area for female and male mice **b)**combined or **c)** separated by sex that received 0 Gy (0Gy) vs. 22 Gy (22Gy) (*n* = 8–10/group). **b)** Data shown as the mean ±SEM for *n* = 8–10 mice/group using an unpaired 2-way Mann-Whitney rank test. **c)** 2-way ANOVA, radiation *p* < 0.001, sex *p* < 0.001, interaction *p* = 0.001, p values obtained from Sidak’s multiple comparison’s test.

To confirm that we had successfully induced cardiotoxicity with this radiation dose, we examined double-stranded DNA (dsDNA) breaks via γH2A.x staining at day 10 post radiation using immunohistochemistry (IHC). Representative images of control 0 Gy vs. 22 Gy are shown in Fig. 2a. Combining females and males, we observed a significant increase in dsDNA breaks in the heart of mice treated with 22 Gy compared to 0 Gy controls (*p* < 0.001) (Fig. 2b). Compared to controls, we found greater dsDNA breaks in females treated with 22 Gy vs. female controls (*p* < 0.001) and in males treated with 22 Gy vs. male controls (*p* = 0.048) (Fig. 2c). By sex, cardiac DNA damage indicated by elevated γH2A.x was greater in females than males (females vs. males *p* < 0.001) (Fig. 2c). 2-way ANOVA revealed significant differences for radiation *p* < 0.0001, sex *p* < 0.001, and an interaction of radiation and sex *p* = 0.001. Thus, acute cardiotoxicity from irradiation was greater in females than males.

#### Radiation induced worse vascular damage in males at day 35

To determine whether cardiac vessels were damaged by 22 Gy irradiation on day 10 and 35, we measured vessel area (%) and vessel wall thickness (VWT). Representative photos of vessels for females (*left*) and males (*right*) (Fig. 3a) are shown using Masson’s trichrome histology stain. Green lines indicate inner and outer vessel tracings used to determine vessel area while 4 lines transecting the vessel were used per vessel to determine vessel wall thickness. At day 10 post radiation, we found increased vessel area in mice treated with 22 Gy vs. controls for females and males combined (*p* < 0.001) (Fig. 3b) and for females (*p* < 0.001) and males (*p* < 0.001) vs. same sex controls (Fig. 3c) (2-way ANOVA, radiation *p* < 0.001, sex *p* = 0.56, interaction *p* = 0.47). Similarly, we found that 22 Gy increased VWT at day 10 compared to controls for females and males combined (*p* < 0.001) (Fig. 3d), and for females (*p* < 0.001) and males (*p* = 0.006) compared to same sex controls (Fig. 3e) (2-way ANOVA, radiation *p* < 0.001, sex *p* = 0.64, interaction *p* = 0.26).

Vessel damage persisted to day 35 after radiation. At day 35 post radiation, we found increased vessel area in mice treated with 22 Gy vs. controls for females and males combined (*p* < 0.001) (Fig. 3f) and for females (*p* < 0.001) and males (*p* < 0.001) vs. same sex controls (Fig. 3g). However, at day 35 post radiation males had larger vessel areas than females (*p* < 0.001) (Fig. 3g) (2-way ANOVA, radiation *p* < 0.001, sex *p* = 0.001, interaction *p* = 0.011). Similarly, we found that 22 Gy increased VWT at day 35 compared to controls for females and males combined (*p* < 0.001) (Fig. 3h). In contrast to day 10, we found that VWT was increased in males vs. females for 0 Gy (*p* = 0.005) and 22 Gy (*p* < 0.001) (Fig. 3i) (2way ANOVA, radiation *p* = 0.88, sex *p* < 0.001, interaction *p* = 0.87). Thus, overall males had both a greater vessel area and greater vessel wall thickness than females at day 35 after radiation.

#### Males develop cardiac hypertrophy by day 10 that persists until day 35

**Figure 4:**
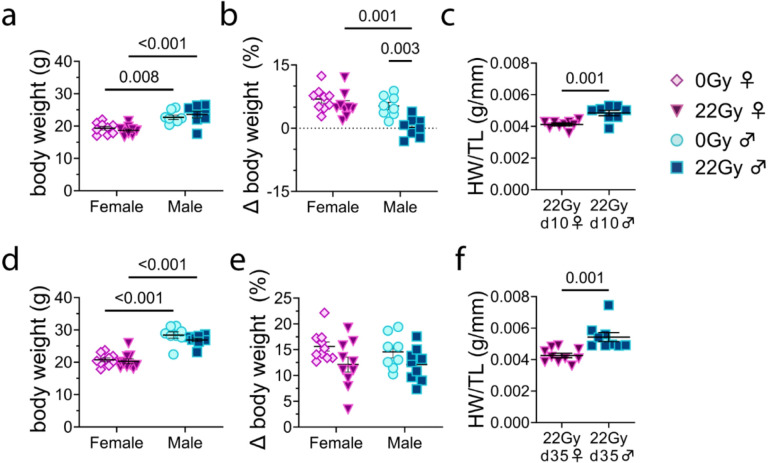
Males develop cardiac hypertrophy. BALB/c female and male mice received 0 Gy (0Gy) vs. 22 Gy (22Gy) irradiation at day 0 and mice were assessed at **a-c)** day 10 (d10) and **d-f)** 35 (d35) for body weight, change in body weight, and heart weight (HW) to tibia length (TL) (i.e., hypertrophy). Data shown as mean ±SEM for 8–10 mice/group. **a,b,d,e)** 2-way ANOVA, p values obtained using Sidak’s multiple comparison’s test. **c,f)**P values obtained using unpaired Mann-Whitney rank tests.

To further determine whether sex differences existed in cardiotoxicity after 22 Gy exposure, we examined body weight (BW) to tibia length (TL) ratio, which is a measure of cardiac hypertrophy. At day 10, males weighed more than females (0 Gy *p* = 0.008, 22 Gy *p* < 0.001) (Fig. 4a) (2-way ANOVA, radiation *p* = 0.044, sex *p* < 0.001, interaction *p* = 0.28). At day 10 males that received 22 Gy lost more body weight than male 0 Gy controls (*p* = 0.003) or 22 Gy females (*p* = 0.001) (Fig. 4b) (2-way ANOVA, radiation *p* = 0.001, sex *p* < 0.001, interaction *p* = 0.053). At day 10, males exposed to 22Gy had a significantly greater BW/TL ratio compared to females (*p* = 0.001) (Fig. 4c), indicating that males developed cardiac hypertrophy by day 10 following radiation exposure while females did not.

At day 35, males continued to weigh more than females (0 Gy *p* < 0.001, 22 Gy *p* < 0.001) (Fig. 4d) (2-way ANOVA, radiation *p* = 0.18, sex *p* < 0.001, interaction *p* = 0.48). But in contrast to day 10, at day 35 males exposed to 22 Gy did not lose more weight than controls (Fig. 4e). Like day 10, at day 35 males exposed to 22Gy continued to have a significantly greater BW/TL ratio compared to females (*p* = 0.001) (Fig. 4f), indicating that cardiac hypertrophy persisted to day 35 after radiation exposure in males.

#### Males develop dilated cardiomyopathy by day 35

**Figure 5:**
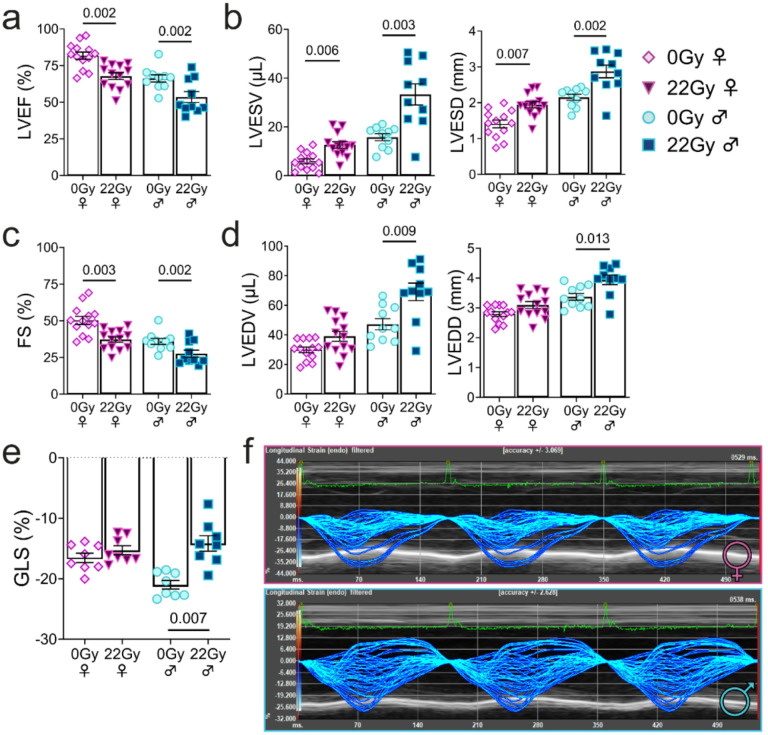
Males develop DCM by day 35. The cardiac function of female and male BALB/c mice was assessed at day 0 (baseline, 0Gy) and 35 after receiving 22 Gy (22Gy) for the following parameters: **a)** left ventricular (LV) ejection fraction (LVEF), **b)**LV end systolic volume (LVESV) and diameter (LVESD), **c)** fractional shortening (FS), **d)** LV end diastolic volume (LVEDV) and diameter (LVEDD), **e)** global longitudinal strain (GLS) (*left*) and **f)** desynchrony using speckle tracking (blue lines) of 48 locations of the endocardial wall inside of the LV over three cardiac cycles in females (*top*) and males (*bottom*) at day 35. **a-e)**Data show the mean ±SEM for *n* = 8–10 mice/group, p values obtained using Wilcoxon matched-pairs signed rank test.

Because of the high variability in heart size and cardiac function observed in normal female and male BALB/c mice, [31] we performed a baseline echocardiography (echo) at day − 1 before irradiation. After irradiation, we performed paired analyses comparing baseline 0 Gy to 22 Gy for each mouse using Wilcoxon matched-pairs rank test by sex (not between sexes) but graphed sexes together. At baseline (day − 1), female and male mice displayed normal ventricular function using echo (Fig. 5). After 22 Gy irradiation, both females and males had a significant reduction in left ventricular (LV) ejection fraction (LVEF) at day 35 compared to baseline (*p* = 0.002, *p* = 0.002; respectively) (Fig. 5a). However, only males had an average drop in LVEF ≤ 50% suggesting heart failure (HF) with reduced EF (HFrEF) while females averaged an LVEF around 65% suggesting HF with preserved EF (HFpEF) (Fig. 5a).

Females and males also developed significant increases in LV end systolic volume (LVESV) (*p* = 0.006, *p* = 0.003; respectively) and diameter (LVESD) (*p* = 0.007, *p* = 0.002; respectively) following irradiation (Fig. 5b), indicating dilatation. Fractional shortening (FS) was also decreased in females and males after irradiation (*p* = 0.003, *p* = 0.002; respectively) (Fig. 5c), indicating a reduced ability to pump blood (i.e., contract). Importantly, only males had a significant increase in LV end diastolic volume (LVEDV) (*p* = 0.009) and diameter (LVEDD) (*p* = 0.013) (Fig. 5d), indicating that males developed DCM by day 35 after 22 Gy exposure.

Global longitudinal strain (GLS) is a sensitive indicator of cardiac stress (i.e., difficulty pumping) in mice and humans, with a percentage of −17 to −20% considered to be normal [31–33]. Thus, after irradiation only males had significantly worse GLS (*p* = 0.007) (closer to 0) compared to baseline (Fig. 5e). Radiation exposure also led to a loss of synchronous cardiac contractions, which can be visualized in the strain curves in Fig. 5f, with females having more synchrony (blue strands closer together) (*top*) than males (separate blue strands) (*bottom*) at 22 Gy. Thus, male BALB/c mice given 22 Gy developed more cardiac strain, HFrEF and DCM by day 35 after irradiation while females have a profile more consistent with HFpEF.

### Radiation leads to cardiac inflammation in males by day 35

To determine whether radiation damage to the heart led to cardiac inflammation, we assessed whether there was a cardiac inflammatory cell infiltration using histology and qPCR in male and female mice that received 0 or 22 Gy. Representative photos of H&E staining to detect inflammatory cells are shown at day 35 in Fig. 6a. Cardiac infiltration was not observed in heart sections of female mice but were observed in males (clumps of purple nuclei, *arrow*) (Fig. 6a).

To determine whether cardiac inflammation changed over time, we examined CD45 levels in the heart using qPCR at 24 hours, day 10 and 35 in females and males (timepoints analyzed on the same RT plate) (Fig. 6b). Female mice had a significant decrease in cardiac CD45 expression at 24 hours post radiation (*p* = 0.025) (Fig. 6b, *left*). In contrast, males had no change in cardiac CD45 expression until day 35 when levels significantly increased (*p* = 0.003) (Fig. 6b, *right*). No increase in inflammation was observed at day 35 in females.

Next, we characterized the cardiac immune infiltrate at day 35 post radiation in females and males using qPCR (sex analyzed on the same RT plate) (Fig. 6c). We found that resident cardiac CD45 + immune cells were present at higher levels at 0 Gy in males than females (*p* = 0.006). However, CD45, a marker of lymphocytes, was significantly increased in males at 22 Gy vs. male controls (*p* = 0.006). Thus, overall inflammation in the heart was increased in males vs. females at 22 Gy (*p* < 0.001) (2-way ANOVA, radiation *p* = 0.048, sex *p* < 0.001, interaction *p* = 0.004). qPCR of CD11b, which is expressed on neutrophils, macrophages, some dendritic cells and mast cells, revealed increased expression in males with radiation vs. controls (*p* = 0.005). Overall, CD11b+ cells were significantly increased in irradiated males vs. females at day 35 (*p* < 0.001) (2-way ANOVA, radiation *p* = 0.048, sex *p* < 0.001, interaction *p* = 0.004). We found that resident F4/80 + cells, indicative of macrophages, were significantly increased in males vs. female controls at day 35 (*p* < 0.001). Macrophages were also increased in males vs. male controls (*p* < 0.001). Macrophages were also expressed at higher levels in the heart of males vs. females at day 35 after radiation (*p* < 0.001) (2-way ANOVA, radiation *p* = 0.028, sex *p* < 0.001, interaction *p* = 0.001). We found that CD4 + T cells were present more often in the heart of control males than females at day 35 (*p* < 0.001). Overall, CD4 + T cells were increased in the heart of males at day 35 compared to females after 22 Gy irradiation (*p* < 0.001) (2-way ANOVA, radiation *p* = 0.62, sex *p* < 0.001, interaction *p* = 0.61). We found that resident cardiac mast cells (based on cKit expression) were present more often in the heart of control males than females at day 35 (*p* < 0.001). Overall, mast cells were increased in the heart of males at day 35 compared to females after 22 Gy irradiation (*p* < 0.001) (2-way ANOVA, radiation *p* = 0.48, sex *p* < 0.002, interaction *p* = 0.028). Additionally, we found that B cells (CD19), CD3 (all T cells), and CD8 (cytolytic T cells) were also increased in males at day 35 after radiation (data not shown). Thus, we found that a mixed infiltrate is present in the heart by day 35 after radiation in males but not in females vs. controls. These data combined with the echo findings indicate that males developed inflammatory DCM (iDCM) following irradiation.

#### Radiation induces fibrosis at day 35 in males

**Figure 7:**
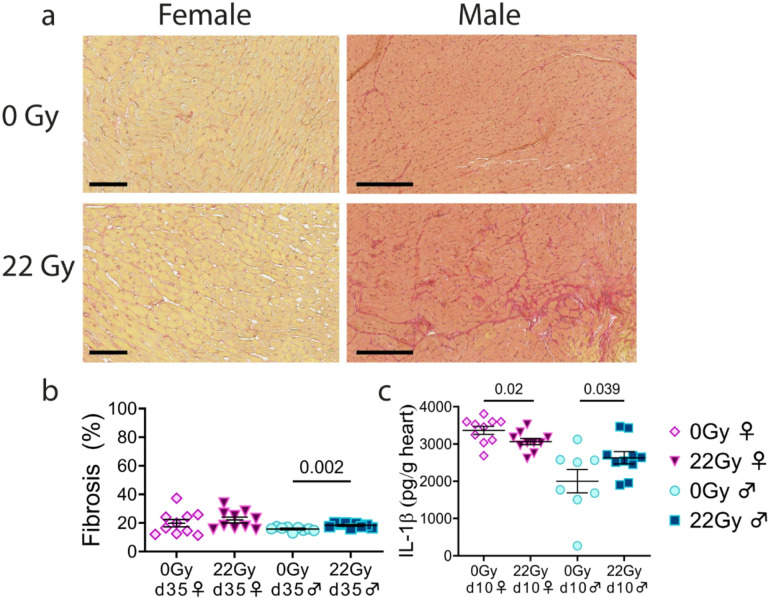
Radiation induces remodeling and fibrosis at day 35 in males. Female and male BALB/c mice received 0 Gy (0Gy) or 22 Gy (22Gy) at day 0 and were examined at day 35 (d35) after irradiation for fibrosis and cardiac cytokine levels by ELISA. **a)**Representative images of collagen deposition in the myocardium using Sirius red stain (Bar = 70μm). **b)** Quantification of fibrosis from Sirius red positive staining normalized to the size of the heart using the overall percentage of positively stained tissue. **c)** ELISA of IL-1β levels in the heart at day 10 after radiation normalized to heart weight. Data shows the mean ±SEM for 8–10 mice/group using 2-way ANOVA, p values obtained using Sidak’s multiple comparison’s test.

To determine whether radiation exposure led to fibrosis on day 10 or 35 after 22 Gy exposure, hearts were stained with picrosirius red to detect collagen deposition. We did not find an increase in collagen deposition at day 10 in the heart of females or males compared to controls (data not shown). Histologically we observed collagen deposition surrounding vessels (not shown), the pericardium (not shown) and the myocardium (Fig. 7a). Representative images of picrosirius staining for collagen deposition (red staining indicates collagen) are shown in Fig. 7a. Quantification of picrosirius red staining using imaging normalized to heart section size found significantly increased collagen levels in the heart of males with 22 Gy compared to controls at day 35 (*p* = 0.002) and a lot more background staining (red rather than yellow background) (Fig. 7b).

The cytokine interleukin (IL)-1β is an early (i.e., acute, day 10) driver of cardiac fibrosis after tissue damage that displays sex differences [30]. For this reason, we examined whether sex differences existed in IL-1β levels in the heart by ELISA of homogenized heart supernatant during acute cytotoxicity following radiation. We found that females had reduced IL-1β levels in the heart at day 10 after radiation (*p* = 0.02), while males had increased levels (*p* = 0.039) (Fig. 7c) (unpaired t-test). IL-1β is produced from both cardiomyocytes and immune cells (i.e., macrophages, mast cells). Thus, the presence of more macrophages and mast cells in males following radiation may increase cardiac IL-1β levels leading to greater fibrosis in males at day 35.

##### RNA sequencing of the heart reveals radiation-induced mitochondrial dysfunction in females and males at day 10

To better understand mechanisms that drive chronic cardiotoxicity in females and males following radiation exposure, we conducted bulk RNA sequencing (RNAseq) on the heart comparing control 0 Gy vs. 22 Gy females and males at day 10 and 35 in separate experiments. Differential gene expression analysis revealed that there were not many significantly different gene changes at day 35 (data not shown), indicating that DCM at day 35 is a cardiac functional endpoint with virtually no gene activity similar to our previous findings [30]. We initially conducted bulk RNAseq comparing males to control males at 22 Gy and later conducted the same analysis for females, so for this reason we cannot directly compare RNAseq between females and males.

Analysis of RNAseq at day 10 using Weighted Gene Co-Expression Network Analysis (WGCNA) (Fig. 8) revealed that the most highly positively correlated gene module in females at day 10 related to radiation was the blue module (Fig. 8a). A strong positive correlation between blue module membership and gene significance was observed (correlation = 0.91, *p* < 1e-131) (data not shown). For the blue module in females, significant enrichment was observed in Gene Ontology (GO) pathways related to cellular structure and organization including “mitotic G1/S transition checkpoint signaling”, “positive regulation of cysteine-type endopeptidase activity involved in apoptotic process”, and “neural tube development”, for example (data not shown). These pathways suggest an increase in structural and signaling adaptation within the heart in females, likely as a response to radiation-induced stress or damage.

The most significantly negatively correlated gene module in females at day 10 was the yellow module (Fig. 8a), which also had a significant correlation (correlation = 0.56, *p* < 9e-12) (Fig. 8b). The top 10 GO pathways enriched in the yellow module all included mitochondrial metabolic pathways such as “oxidative phosphorylation”, “aerobic respiration,” and “proton-motive force driven ATP synthesis”, for example, suggesting that mitochondrial homeostasis was significantly impaired by radiation exposure at day 10 in females (Fig. 8c).

Analysis of the RNAseq using WGCNA in males revealed that the most highly positively correlated gene module in males at day 10 related to radiation was the blue module (Fig. 8d). A strong positive correlation between blue module membership and gene significance was observed (correlation = 0.55, *p* < 1e-200) (data not shown). For the blue module in males, significant enrichment was observed in GO pathways related to cellular structure and organization including “establishment of organelle localization”, “cilium organization”, and “actin filament-based process regulation”, for example (data not shown). These pathways suggest an increase in structural and signaling adaptation within cells in males, likely as a response to radiation-induced stress or damage.

The most significantly negatively correlated gene module in males at day 10 was the pink module (Fig. 8d), which also had a significant correlation (correlation = 0.54, *p* < 1e-50) (Fig. 8e). The top 10 GO pathways enriched in the pink module all included mitochondrial metabolic pathways such as “energy derivation by oxidation of organic compounds”, “cellular respiration”, and “oxidative phosphorylation” (Fig. 8f), suggesting that mitochondrial homeostasis was significantly impaired at day 10 after radiation exposure in males. Thus, the gene profile at day 10 revealed upregulated cardiac repair mechanisms and dysregulation (i.e., downregulation) of mitochondrial pathways in both sexes following radiation exposure.

#### Females upregulate Pgc1α at day 10 after radiation indicating mitochondrial repair

**Figure 9:**
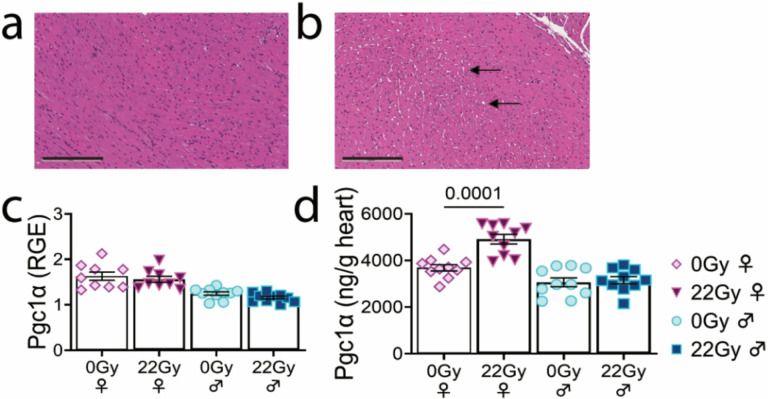
Radiation induces mitochondrial damage which is improved in females. Male and female BALB/c mice received 0 or 22 Gy radiation on day 0 and hearts analyzed at day 10 post radiation. Representative histology photos using H&E in **a)**0 Gy vs. **b)** 22 Gy hearts displaying greater vacuolization (white spaces, arrows) after radiation (Bar = 60μm). **c)** Relative gene expression of *Pgc1α* levels normalized to *Hprt*in the heart at day 10 post radiation. **d)** ELISA of Pgc1α levels in the heart of homogenized hearts normalized to weight at day 10 after radiation.

We observed mitochondrial damage following radiation in females and males compared to controls based on cardiac vacuolation observed using H&E histology. Representative images of the heart showing occasional vacuoles (Fig. 9a) in controls vs. dense areas of vacuolization (Fig. 9b) are shown following 22 Gy at day 10.

Pgc1α is a master regulator of mitochondrial function which improves mitochondrial function when it is elevated [34–36]. Although mitochondrial damage occurs following irradiation in both sexes as indicated by RNAseq, we found that cardiac protein levels of Pgc1α by ELISA are upregulated in in the heart of females at day 10 (*p* = 0.001) but not in males (Fig. 9d) suggesting that females are better able to repair mitochondrial damage to the heart following radiation.

## Discussion

**Figure 10:**
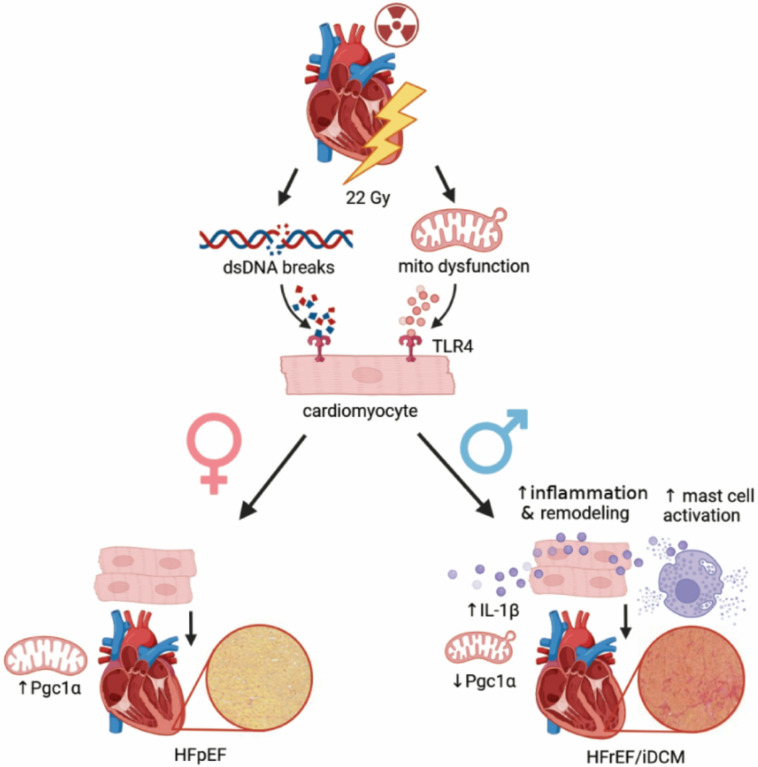
Cartoon illustrating proposed mechanisms of sex differences in radiation-induced cardiotoxicity. Radiation directed to the heart leads to dsDNA breaks, mitochondrial dysfunction and vacuolation by day 10 after radiation. Damage to the heart upregulates TLR4 which leads to IL-1β production in the heart. Females repair mitochondrial dysfunction by upregulating the master regulator of mitochondrial function, Pgc1α. This results in less damage, no elevation in the profibrotic cytokine IL-1β, no fibrosis and less chronic cardiac dysfunction represented as HFpEF. In contrast, males do not upregulate Pgc1α in response to damage resulting in recruitment of inflammatory cells to the heart, elevated IL-1β resulting in fibrosis, iDCM and HFrEF by day 35 after radiation.

This study is the first, to our knowledge, to examine sex differences in RIHD in a translational mouse model. The main findings of this study were that radiation exposure to the heart led to cardiac dsDNA breaks, mitochondrial dysfunction, vacuolation and vessel hypertrophy by day 10 in both males and females (Fig. 10). Mitochondrial damage releases ATP and other mitochondrial components that activate TLR4 leading to IL-1β production [37, 38]. Females repaired mitochondrial dysfunction by upregulating the master regulator of mitochondrial function, Pgc1α. This resulted in less cardiac damage, IL-1β, fibrosis and better cardiac function (i.e., HFpEF). In contrast, males did not upregulate Pgc1α in response to mitochondrial damage resulting in recruitment of inflammatory cells to the heart, elevated IL-1β, fibrosis, iDCM and HFrEF after radiation (Fig. 10).

Radiation is known to cause dsDNA breaks, [39, 40] and so we examined this outcome as evidence that we had induced radiation damage in the heart. Surprisingly, we found that females had a higher number of dsDNA breaks based on acute γH2AX expression in the heart after 22 Gy irradiation. Studies suggest that women may be at significantly greater risk of suffering and dying from radiation-induced cancer than men exposed to the same dose of radiation [41]. A study of peripheral blood mononuclear cells (PBMCs) from healthy volunteers found no difference in γH2Ax expression by sex [42]. Similarly, a study by Fernandez et al. examined γH2Ax expression in PBMCs in patients with bladder cancer but did not observe sex differences [43]. It is unclear why we found higher γH2AX expression in the heart of females at day 10 but one possibility is that DNA repair mechanisms differ by sex. Mayer et al. found that DNA in cells from men rejoin at a higher percentage of ds breaks than cells from women [44].

Radiation exposure is also known to induce mitochondrial dysfunction [4]. Analysis of bulk RNAseq from whole hearts in this study found that both males and females developed mitochondrial dysfunction following 20 Gy irradiation. However, we found elevated levels of Pgc1α in the heart of females after irradiation that was not observed in males. PGC1α is a major regulator of mitochondrial homeostasis because it regulates the synthesis of mitochondrial proteins. PGC1α was originally identified as a regulator of mitochondrial function in brown adipose tissue but was later also found to be expressed at high levels in cardiac tissue where it influences cardiovascular health and disease [45]. PGC1α globally regulates mitochondrial pathways in response to stresses such as cold, fasting, infection and radiation [36, 46]. Sex differences in mitochondrial function in health and disease are also known [36, 47]. Naumenko et al. showed in 2022 that cardiac-specific Pgc1α deficiency led to heart failure that occurred more quickly and was more severe in females than males, suggesting a greater protective effect of Pgc1α in females [47]. Estrogen (i.e., 17β-estradiol) has been shown to regulate PGC1α expression in the heart [48]. We previously found that females with viral myocarditis had higher expression of PGC1α in their hearts than males after viral damage to mitochondria that was associated with reduced TLR4/IL-1β, fibrosis and improved cardiac function in females [36]. Thus, the metabolic stress of radiation damage is a likely explanation for the elevated levels of PGC1α that we observed in females after 20 Gy exposure that may have led to their improved cardiovascular outcomes.

Sex differences in inflammation are well described [14, 49]. Females may be protected from many CVDs because the major immune factor that regulates inflammation, forkhead box P3 (FoxP3), is located on the X chromosome [49, 50]. Additionally, elevated 17β-estradiol levels in females further promote the expression of FoxP3- a marker of regulatory T cells (Tregs) [51]. Estrogen also drives a more regulatory phenotype in other immune cells such as macrophages, dendritic cells and T helper (Th)2/IL-4 responses [50, 52]. For example, cardiac inflammation in females with viral myocarditis have elevated anti-inflammatory alternatively activated M2 macrophages, Tim3^+^ CD4 T cells, Tregs and IL-4 [8]. Our findings in this study of an elevated CD11b^+^ and F4/80^+^ infiltrate in males following radiation exposure with elevated IL-1β levels, fibrosis and progression to iDCM is remarkably similar to the cardiac immune profile of males with viral myocarditis, [8, 9] suggesting common pathogenic mechanisms following mitochondrial damage to the heart. Similar sex differences have been observed in patients with myocarditis [53]. Thus, our finding of iDCM in males with radiation exposure is consistent with other mouse models and clinical data. Future research is needed to determine whether RIHD in males is overlooked for more traditional explanations (i.e., diagnoses like atherosclerosis or idiopathic DCM).

### Perspectives and Significance

To our knowledge, this is the first study of sex differences in RIHD in mice. We found that females and males developed cardiotoxicity following radiation exposure, but sex differences were observed in the progression of disease. Mitochondrial dysfunction occurred in both sexes; however, females recovered better by upregulating Pgc1α which is known regulator of mitochondrial homeostasis. This resulted in less cardiac inflammation and fibrosis in females and progression to a phenotype resembling HFpEF, which is known to occur more often in females. In contrast, males progressed to iDCM and HFrEF, conditions that are known to occur more often in males. These findings highlight the clinical challenges in diagnosing RIHD, because it so closely resembles the outcome of other cardiovascular conditions. Mouse models that replicate clinical findings are important tools for identifying early biomarkers and potential therapies to improve outcomes in RIHD.

## Methods

### Animal care and ethics statement

Mice were used in accordance with recommended practices in the Guide for the Care and Use of Laboratory Animals of the National Institutes of Health (NIH), with approval obtained from the Institutional Animal Care and Use Committee (IACUC) at Mayo Clinic Florida for all procedures (IACUC# A00005356–20 and A00006478–22) as well as the Biosafety Committee.

### Mice

Adult female and male BALB/cJ (BALB/c) (strain #000651) 8–10-week-old mice were obtained from the Jackson Laboratory (Bar Harbor, ME) and maintained under pathogen-free conditions in the animal facility at Mayo Clinic Florida. We previously showed that we are powered to detect sex differences in cardiomyopathy with at least 7 mice/group [36, 54]. 7–10 mice per group were used for all experiments, unless otherwise indicated. Experiments were repeated to obtain all endpoints and ensure rigor. Tissues were collected at 24 hours (innate cardiac damage), day 10 (acute cardiotoxicity) or day 35 postirradiation (chronic cardiotoxicity/cardiomyopathy).

### Radiation model

Radiation exposure utilized an X-Rad SmART (Precision X-Ray, Madison, CT) small animal irradiator. Male and female mice were anesthetized using ketamine/midazolam (12.5mg/mL;0.6 mg/mL) and loosely wrapped in sterile pre-warmed fabric to prevent hypothermia inside of the irradiation chamber. Mice were randomly assigned to a group to receive: 0 Gy (control, anesthesia only) or 22 Gy radiation in a single dose. Control (0 Gy) mice received ketamine/midazolam only and were placed in the device for the equivalent time as irradiated mice. Mice were placed in a feet-first prone position in relation to the gantry and radiation was delivered in a single fraction through the axillary region using a 5mm collimator with the isocenter of radiation focused superior to the apex and inferior to the atrial appendages, visualized via computed tomography (CT) scan.

### Histology

Murine cardiac tissue was dissected longitudinally along the sagittal plane at day 10 and day 35 after irradiation and fixed in 10% phosphate-buffered formalin for 48 hours, shielded from light, and embedded in paraffin for histological analysis. 5μm sections were stained with hematoxylin and eosin (H&E) to detect inflammation and vacuolation, Masson’s trichrome or picrosirius red to detect collagen. The number of strong positives (NSP) from scanned cardiac tissues for picrosirius red staining were assessed as a ratio of NSP compared to the total area of the section and quantified, as previously described [55].

### Immunohistochemistry

Embedded cardiac tissue sections were cut at 5μm and stained with γH2A.x on day 1 post radiation (Cell Signaling Technology, Danvers, MA, USA 9718S, 1:5000, rabbit). Envision+ anti-rabbit-labeled polymer (K4003) was used as the secondary antibody. IHC slides were scanned using an Aperio AT2 slide scanner (Leica, Wetzlar, Germany) and quantified as the number of strong positives for γH2A.x divided by the total area of the section to calculate the percentage of positivity, as previously described [55].

### Echocardiography

Cardiac function was measured using transthoracic echocardiography on day − 1 (baseline, 0 Gy) and day 35 (endpoint, 22 Gy) with the Vevo 3100 ultrasound machine equipped with a MX55OD 55 MHz transducer (VisualSonics Inc., Toronto, Canada), as previously described [27, 36]. Anesthesia (12.5mg/mL ketamine; 0.6mg/mL midazolam) was administered by intraperitoneal injection and mice were placed in a supine position on a warming platform, with a rectal thermometer placed to monitor for hypothermia. Hair covering the thoracic region was removed using Nair and pre-warmed ultrasound gel (Parker Laboratories, Fairfield, NJ) was applied to the region. The probe was then placed to encompass the long-axis of the left ventricle and B-mode images and videos were captured for global longitudinal strain (GLS) analysis using Vevo Strain software, as previously [27, 36]. Minimal adjustments were made to the speckle tracking and auto-LV artificial intelligence tracings when necessary and all analysis was performed while blinded to the treatment group. Fractional shortening (FS) is the percent change in LV cavity dimensions. Ejection fraction (EF) represents stroke volume (SV) as a percentage of end diastolic LV volume. The heart rate was determined using the built-in echocardiography probes in the Vevo Lab Imaging Station.

### RNA isolation and qRT-PCR

At harvest, hearts were sectioned longitudinally along the sagittal plane to create two halves, snap frozen, and stored at −80°. Half hearts were later homogenized and lysed using Tissuelyser (Qiagen, Hilden, Germany) with 7mm stainless steel beads in RTL buffer with 0.5% DX buffer to reduce foam. RNA was isolated using Qiagen’s RNase Easy Fibrous Tissue Mini Kit (Qiagen, 74704). RNA concentration (Abs. 260) and quality (Abs. 260/280) were assed using a Nanodrop (Thermo-Scientific, Waltham, MA, 11095–080). RNA was converted to complementary DNA (cDNA) using iScript cDNA synthesis kit (Biorad, Hercules, CA, USA, #1708891), as previously described [36]. Two-step quantitative reverse transcriptase-mediated real-time PCR (qPCR) was used to measure abundance of individual mRNAs. Gene expression was measured using assay-on-demand primers and probe sets and the ABI7000 Taqman system (Applied Biosystems, Foster City, CA). Gene expression was assessed by comparative quantification, as previously [55].

### RNA sequencing

Library preparation and bulk-tissue mRNA sequencing (RNAseq) for male and female control 0 Gy vs. 22 Gy irradiated hearts (*n* = 8–9/group) were performed by the Mayo Clinic Genome Analysis Core. Analysis of the data was performed as previously reported. Libraries for this study used a standard poly A selection. RNA libraries were prepared using 200ng of total RNA according to the manufacturer’s instructions for the TruSeq Stranded mRNA Sample Prep Kit (Illumina, San Diego, CA). The concentration and size distribution of the completed libraries were determined using an Agilent Bioanalyzer DNA 1000 chip (Santa Clara, CA) and Qubit fluorometry (Invitrogen, Carlsbad, CA). Libraries were sequenced at 50 million fragment reads per sample following Illumina’s standard protocol using the Illumina cBot and HiSeq 3000/4000 PE Cluster Kit. The flow cells were sequenced as 100 X 2 paired end reads on an Illumina HiSeq 4000 using HiSeq 3000/4000 sequencing kit and HiSeq Control Software HD 3.4.0.38 collection software. Base-calling was performed using Illumina’s RTA version 2.7.7.

### Data pre-processing

Aligned cram files were generated by the Mayo Clinic Genome Analysis Core from the RNAseq data, which were then processed to quantify gene expression levels. RNAseq read count processing was performed on these cram files using the featureCounts tool from Subread v2.0.3 with the Gencode vM30 annotation GTF. Lowly expressed genes (mRNA count < 10) were filtered out and the remaining counts normalized downstream using DESeq2 v1.44.0. The resulting count matrix was subsequently utilized for downstream differential expression analysis and functional enrichment analysis using appropriate statistical methods.

### Differential gene expression analysis

For differential expression analysis, we utilized edgeR version 4.2.2 on two separate RNA-Seq datasets collected at day 10. Each dataset consisted of gene expression counts across samples from two groups: control (0 Gy) and treatment (22 Gy). Raw count data and corresponding metadata for each day were imported from CSV files. The raw count data contained expression counts for each gene across samples, while the metadata included sample information such as group and time point. Separate DGEList objects were created in edgeR for each dataset (day 10 and 35), encapsulating the count data and group information.

To ensure reliable differential expression analysis, lowly expressed genes were filtered out in each dataset using the filterByExpr function, retaining only genes with adequate counts across samples. After filtering, the DGEList objects were updated, and library sizes were recalculated to exclude lowly expressed genes. Normalization factors were then calculated using the calcNormFactors function to adjust for differences in library sizes and composition across samples, ensuring fair comparisons of expression levels between samples within each dataset. Biological variability across samples was accounted for by estimating dispersion for each gene in both datasets using the estimateDisp function. Dispersion estimation is essential for accurately modeling count data variability, particularly for experiments with biological replicates. Each DGEList object was updated with these dispersion estimates to improve the robustness of downstream analyses. Differential expression testing was then performed separately for each dataset using the exactTest function, which conducts a statistical test for differences in gene expression between the control (0Gy) and treatment (22Gy) groups. This approach is based on the negative binomial distribution and incorporates the dispersion estimates calculated for each dataset. Gene IDs in the original count data were provided as murine Ensembl gene IDs (ENSMUSG). These IDs were converted to gene symbols using the biomaRt package version 2.60.1 in conjunction with the org.Mm.eg.db database version 3.19.1, allowing for easier interpretation and compatibility with annotation databases. The results of the differential expression analysis were extracted using the topTags function, providing a ranked list of genes based on statistical significance for each time point. We also used the decideTests function to generate a summary of significant genes, indicating the number of upregulated and downregulated genes for each dataset. The full differential expression results, including fold changes, p-values, and adjusted p-values (FDR), were saved as separate CSV files for each time point (day 10 and 35). These files contain information on all tested genes, enabling further interpretation and validation of differentially expressed genes. This edgeR workflow allowed us to identify genes with significant expression changes associated with the treatment groups over time, providing insights into the molecular response at each time point.

### Co-expression network analysis

To identify gene co-expression modules and their association with experimental traits, we used the Weighted Gene Co-expression Network Analysis (WGCNA) R package v1.73. We filtered genes to include only those with significant variability across samples. The data were then normalized, and soft-thresholding powers (β) of 3 for day 10 and 4 for day 35 were selected to achieve approximate scale-free topology fits of R^2^ > 0.85 for day 10 and 0.8 for day 35, as determined by the criterion of approximate scale-free topology. Using these selected β values, we transformed the similarity matrices into adjacency matrices, capturing gene connectivity based on co-expression. These were further converted into Topological Overlap Matrices (TOMs) to enhance robustness by incorporating shared connections between genes. Gene modules were identified by hierarchical clustering on the TOM-based dissimilarity, followed by dynamic tree cutting to delineate distinct modules, with each module assigned a unique color for easy visualization. To explore the relationship between modules and the trait of interest, we computed module eigengenes (the first principal component of each module) to summarize the expression profile of each module. We then correlated these module eigengenes with the external trait (“Radiation”), identifying modules significantly associated with the trait. These relationships were visualized in a heatmap, where significant modules exhibited strong positive or negative correlations. For modules with significant correlations, we created scatter plots of module membership (correlation between each gene and the module eigengene) against gene significance (correlation between each gene and the trait) to assess the strength of association between module relevance and trait significance. To interpret the biological relevance of each gene module, we conducted pathway enrichment analysis using the Gene Ontology (GO) database. Genes from each module were extracted and converted to gene symbols or Entrez IDs, as required for pathway analysis tools, and only genes from modules significantly correlated with the trait were included in the enrichment analysis. The clusterProfiler R package v4.12.6 was employed to test for the overrepresentation of GO terms (Biological Process) in each module. This analysis used a hypergeometric test, excluding GO terms with fewer than 5 or more than 500 genes to avoid non-specific results. P-values from the enrichment analysis were adjusted for multiple testing using the Benjamini-Hochberg procedure, with a false discovery rate (FDR) threshold of 0.05 for significance. For each significant module, the top 10 enriched pathways were visualized in bar plots using ggplot2 v3.5.1, where bar lengths represented the number of genes associated with each pathway and colors indicated the level of statistical significance (p-value). Pathways with significant enrichment (FDR < 0.05) were interpreted to provide insights into the biological functions associated with each module. This workflow enabled the identification of biologically relevant gene modules and the elucidation of their functional roles through pathway enrichment, offering insights into the underlying molecular processes associated with the trait of interest.

### Vessel measurements

Vessel wall thickness (VWT) was determined from H&E-stained slides by measuring the width of 3 vessels/sample using ImageScope at high magnification at 4 locations for each vessel. Vessel area (%) was obtained by taking the area of the outer lumen minus the area of the inner lumen x100 using ImageScope of 3 vessels/sample.

### ELISA

Frozen hearts were rapidly thawed and weighed to obtain tissue wet weight before homogenizing using a polytron homogenizer in minimum essential media (MEM) with 2% fetal bovine serum (FBS). Homogenized tissue was centrifuged at 3,000 rpm at 6°C for 20 minutes and the supernatant was collected for analysis. Protein expression was quantified using the Mouse Estrogen-Related Receptor Alpha ELISA Kit (MyBioSource, cat#MBS080310, San Diego, CA). Absorbance was used to calculate concentration relative to a standard curve and normalized to tissue wet weight, as previously [55–58]. The lowest detection limit for the ERRα kit was 0.1 ng/mL with a detection range of 0.25–8 ng/mL.

### Statistical Analysis

Normally distributed data comparing two groups were analyzed using a 2-tailed Student’s *t* test. The Mann-Whitney rank sum test was used to evaluate nonparametric data. Data are expressed as mean ± SEM. When comparing more than 2 groups multiple comparison analysis was performed by ANOVA (and nonparametric equivalent for nonparametric data) with each group compared to the corresponding control group; 2-way ANOVA examined the effect of radiation and sex. We previously showed that we are powered to detect sex differences in cardiac diseases with at least 7 mice/group [36, 54]. 7–10 mice per group were used for all experiments, unless otherwise indicated. Data are expressed as mean ± SEM. A value of *p* < 0.05 was considered significant.

## Figures and Tables

**Figure 3:** Radiation induces vascular damage at day 10 and 35 in females and males. **a)**Representative photos of vessels for females (pink) and males (blue) are shown using Masson’s trichrome stain. Green lines indicate inner and outer vessel tracings used to determine vessel area while 4 lines were used/vessel to determine vessel wall thickness (VWT). Female and male BALB/c mice received 0 Gy (0Gy) or 22 Gy (22Gy) at day 0 and were examined at **b-e)** day 10 (d10) and **f-i)** day 35 (d35) after irradiation for vascular damage. Vessel area and VWT are shown for males and females combined (b,d,f, h) or separated by sex (c,e,g,i). Data show the mean ±SEM for 8–10 mice/group. **b,d,f,h)**P values obtained using unpaired Mann-Whitney rank tests. **c,e,g,i)** 2-way ANOVA, p values obtained using Sidak’s multiple comparison’s test.

**Figure 6:** Radiation induces cardiac inflammation at day 35 in males. **a)** Representative H&E histology are shown for females and males at day 35 after irradiation (bar = 60μm). Arrow indicates inflammation. **b)** Female (pink) and male (blue) BALB/c mice received 0 (0Gy) (grey) or 22 (22Gy) Gy on day 0 and hearts examined at 24 hours (24H), day 10 (d10) or day 35 (d35) after irradiation by qPCR for relative gene expression (RGE) of CD45 lymphocytes normalized to the housekeeping gene *Hprt*. **c)** Hearts at day 35 after irradiation were analyzed for immune cells by qPCR for CD45 (lymphocytes), CD11b (neutrophils, macrophages, some dendritic cells, mast cells), F4/80 (macrophages), CD4 T helper cells, and cKit mast cells. **b,c)** Data shown as mean ±SEM for 5–10 mice/group. **c)** 2-way ANOVA, p values obtained using Sidak’s multiple comparison’s test.

**Figure 8:** RNA sequencing reveals radiation-induced mitochondrial dysfunction in females and males at day 10. **a-c)** Female and **d-f)** male BALB/c mice received 0 or 22 Gy radiation on day 0 and bulk RNA sequencing performed on hearts analyzed at day 10 post radiation (*n* = 8–9/group). **a,d)** Module color assignments from high dimensional Weighted Gene Co-Expression Network Analysis reveal top up- (red) or down(blue) regulated genes comparing 0 to 22 Gy by sex. Significantly down-regulated gene modules that relate to mitochondrial function for **b)** females (yellow module, correlation = 0.56, *p* = 9.3 e-12) and **e)** males (pink module, correlation = 0.54, *p* = 5.1 e-50). Modules represent clusters of co-expressed genes associated with distinct transcriptional programs. Top 10 significant changed gene ontology (GO) pathways related to mitochondrial function in the **c)** yellow module in females and **f)** the pink module in males.

## Data Availability

Data used for RNA sequencing and subsequent WGCNA are available upon request. Other requests for data used in this manuscript can be addressed to the corresponding author and are available upon reasonable request.

## Highlights

- First translational study demonstrating sex differences in RIHD.
- Cardiac DNA damage, mitochondrial dysfunction, vacuole formation, and increased vessel size occurred in both sexes after radiation exposure.
- Males developed worse RIHD than females with elevated IL-1β, cardiac remodeling and inflammatory DCM.
- Females improved recovery after mitochondrial damage by upregulating Pgc1α leading to mild cardiomyopathy following radiation.

## Abbreviations

ANOVA: analysis of variance
BW: body weight
CT: computed tomography
CVDs: cardiovascular diseases
DCM: dilated cardiomyopathy
ds: double-stranded
Echo: echocardiography
ELISA: enzyme linked immunosorbent assay
FoxP3: forkhead box P3
FS: fractional shortening
GLS: global longitudinal strain
GO: Gene Ontology
Gy: Gray
H&E: hematoxylin and eosin
WGCNA: Weighted Gene Co-Expression Network Analysis
HF: heart failure
HFpEF: heart failure with preserved ejection fraction
HFrEF: heart failure with reduced ejection fraction
IACUC: Institutional Animal Care and Use Committee
iDCM: inflammatory dilated cardiomyopathy
IHC: immunohistochemistry
IL: interleukin
LV: left ventricular
LVEDD: left ventricular end diastolic diameter
LVEDV: left ventricular end diastolic volume
LVEF: left ventricular ejection fraction
LVESD: left ventricular end systolic diameter
LVESV: left ventricular end systolic volume
NIH: National Institutes of Health
NSP: number of strong positives
PBMCs: peripheral blood mononuclear cells
qPCR: quantitative RT-PCR
RIHD: radiation-induced heart disease
RNAseq: RNA sequencing
Th: T helper
TL: tibia length
TLR4: Toll-like receptor 4
Tregs: regulatory T cells
VWT: vessel wall thickness
